# Supplementary material for: Spatial transcriptomics reveals unique gene expression changes in different brain regions after sleep deprivation
Source: Nat Commun. 2023 Nov 4;14:7095. doi: 10.1038/s41467-023-42751-z (PMC10625558; doi:10.1038/s41467-023-42751-z)
Supplement: Supplementary file 1 — Supplementary Information [file 41467_2023_42751_MOESM1_ESM.pdf]

**Supplementary Information for**

# Spatial transcriptomics reveals unique gene expression changes in different brain regions after sleep deprivation

Yann Vanrobaeys[1,2,3], Zeru J. Peterson[2,5], Emily. N. Walsh[2,3,4], Snehajyoti Chatterjee[2,3], Li-Chun Lin[2,3,7], Lisa C. Lyons[6], Thomas Nickl-Jockschat[2,3,5*], Ted Abel[2,3*]

[1]Interdisciplinary Graduate Program in Genetics, University of Iowa, 357 Medical Research Center Iowa City, IA 52242, USA

[2]Iowa Neuroscience Institute, Carver College of Medicine, 169 Newton Road, 2312 Pappajohn Biomedical Discovery Building, University of Iowa, Iowa City, IA 52242, USA

[3]Department of Neuroscience and Pharmacology, Carver College of Medicine, 51 Newton Road, 2-417B Bowen Science Building, University of Iowa, Iowa City, IA 52242, USA

[4]Interdisciplinary Graduate Program in Neuroscience, University of Iowa, 356 Medical Research Center, Iowa City, IA 52242, USA

[5]Department of Psychiatry, University of Iowa, Iowa City, IA, USA

[6]Program in Neuroscience, Department of Biological Science, Florida State University, Tallahassee, FL, USA

[7]Department of Neurology, University of Iowa, Iowa City, IA, USA

* correspondence to ted-abel@uiowa.edu or thomas-nickl-jockschat@uiowa.edu

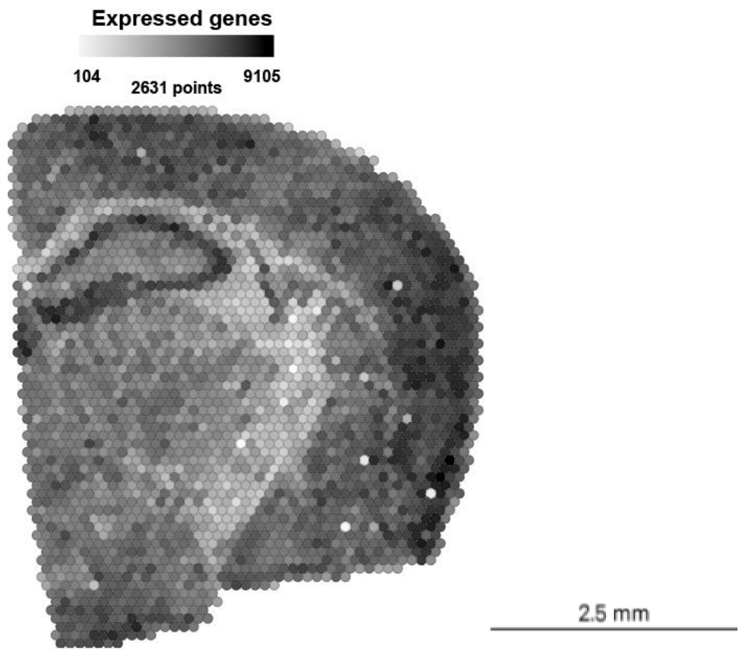


**Sup. Fig. 1. Number of expressed genes across the tissue section of sample 5.** The darkness of the spot reflects the number of expressed genes detected. The highest numbers of genes are detected where spots overlap the location of excitatory neurons in the cortex and pyramidal neurons of the hippocampus.

Sample 1

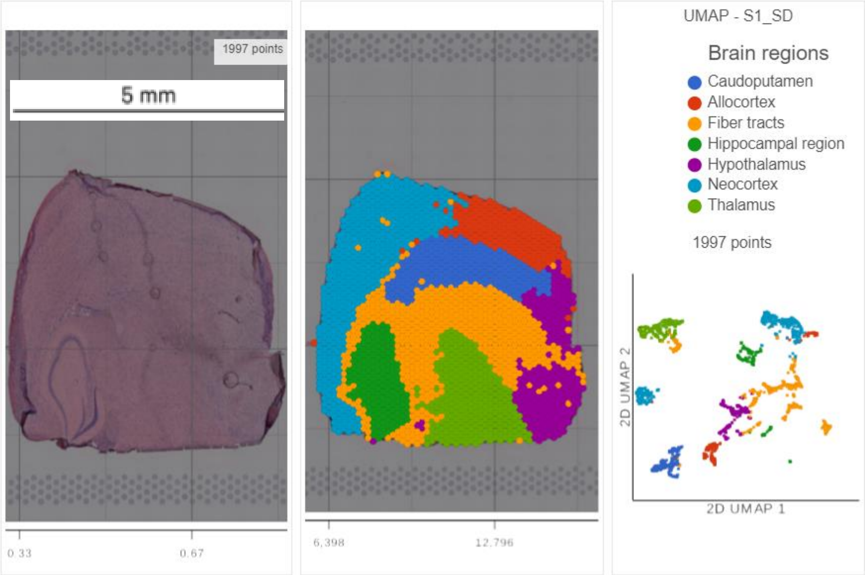


Sample 2

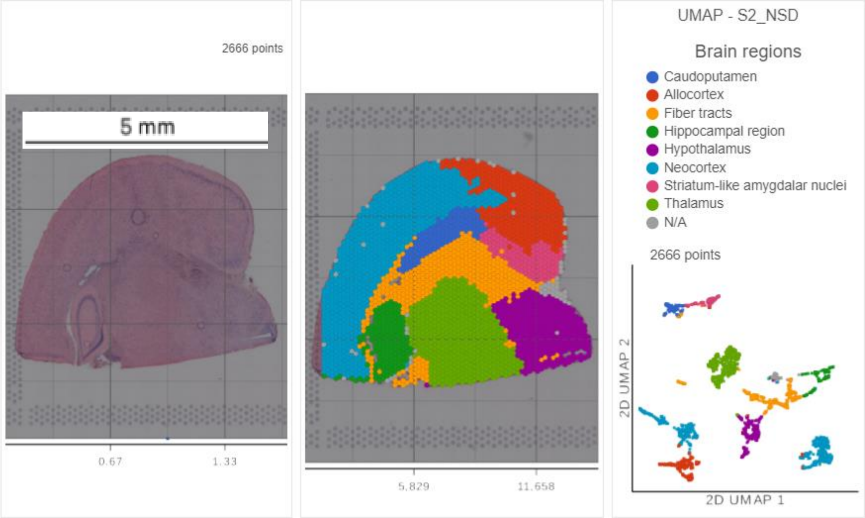

Sample 3

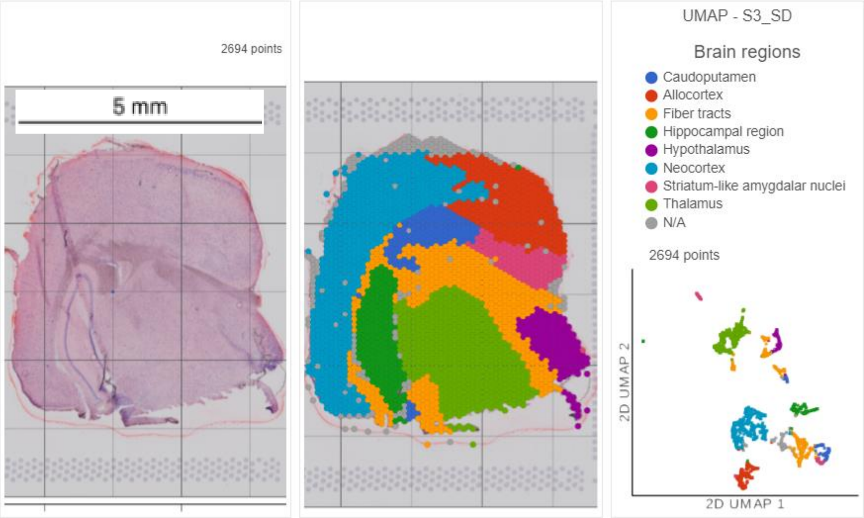


Sample 5

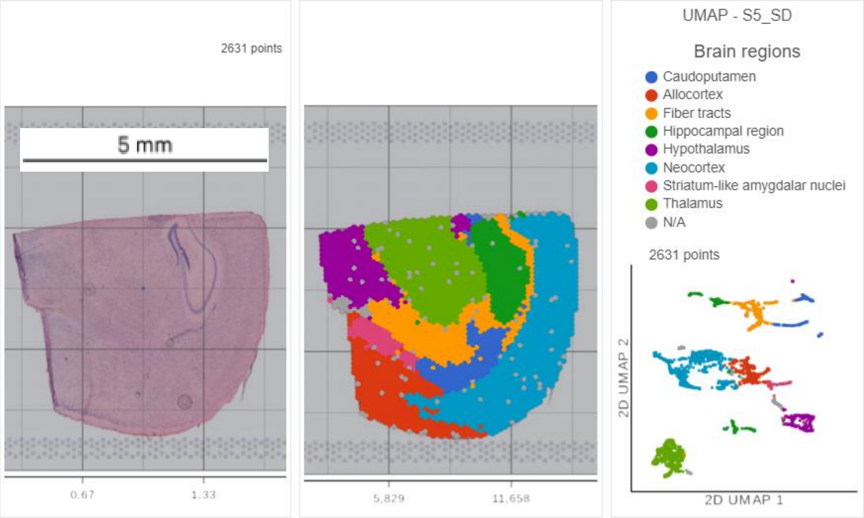


Sample 6

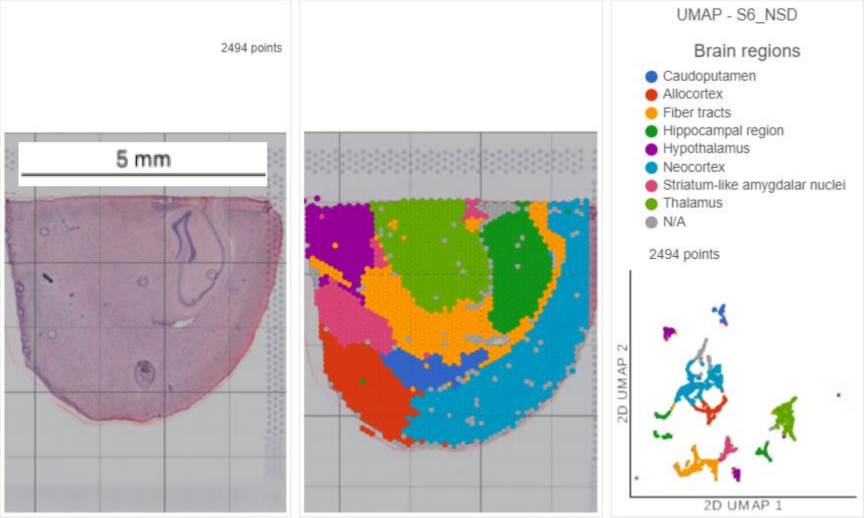

Sample 7

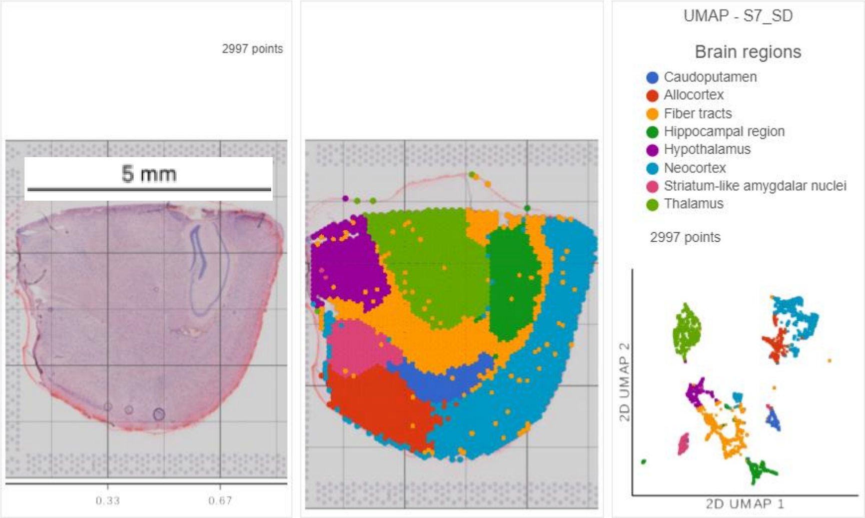


Sample 8

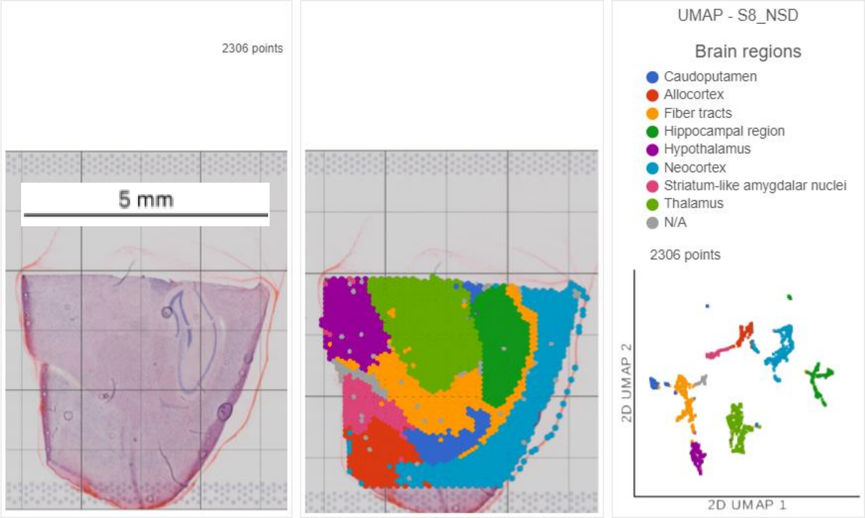


Sample 9

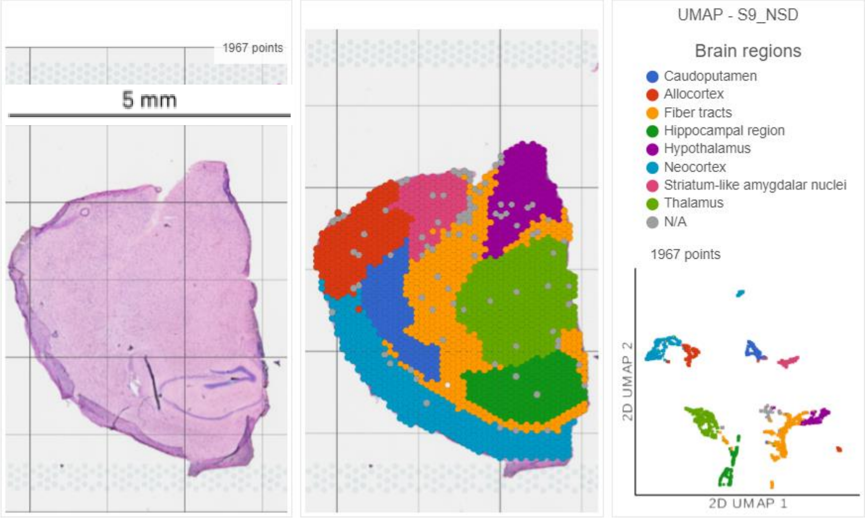

Sample 10

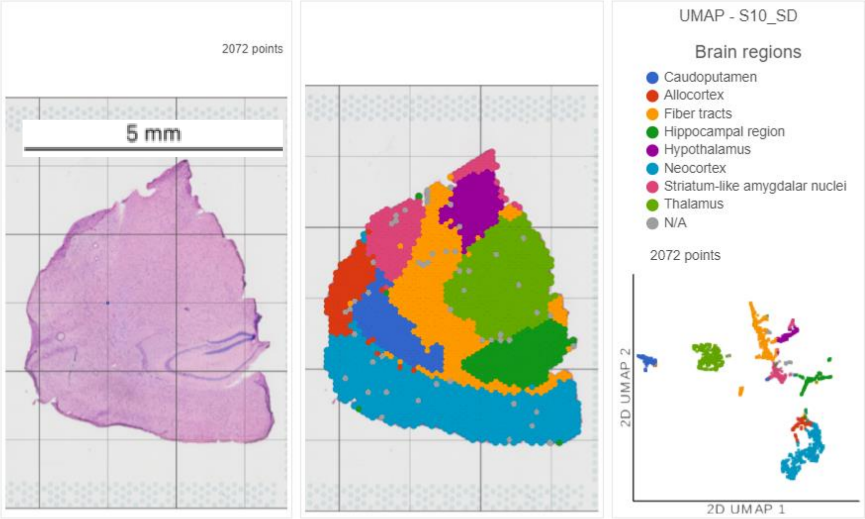


Sample 11

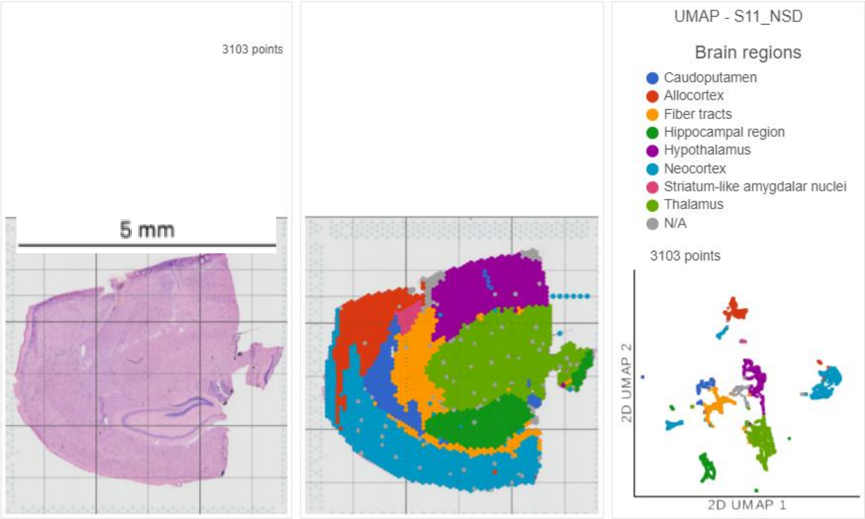


Sample 12

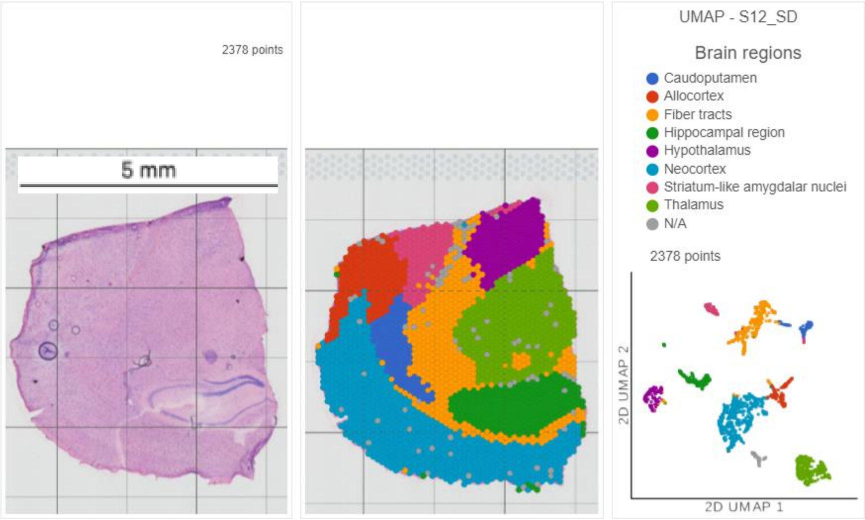

Sample 13

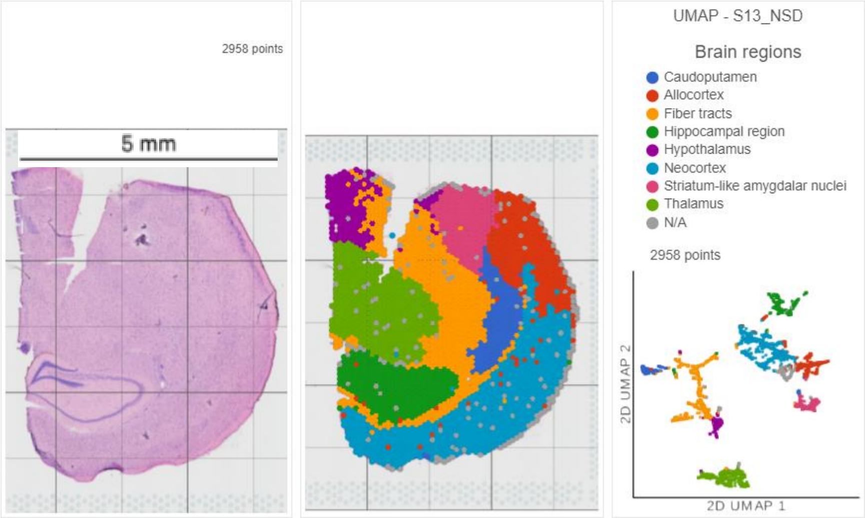


Sample 14

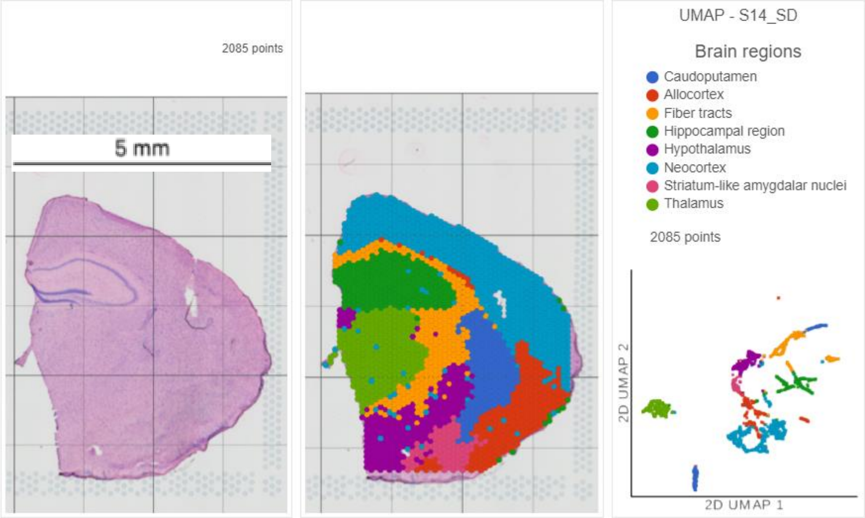


Sample 15

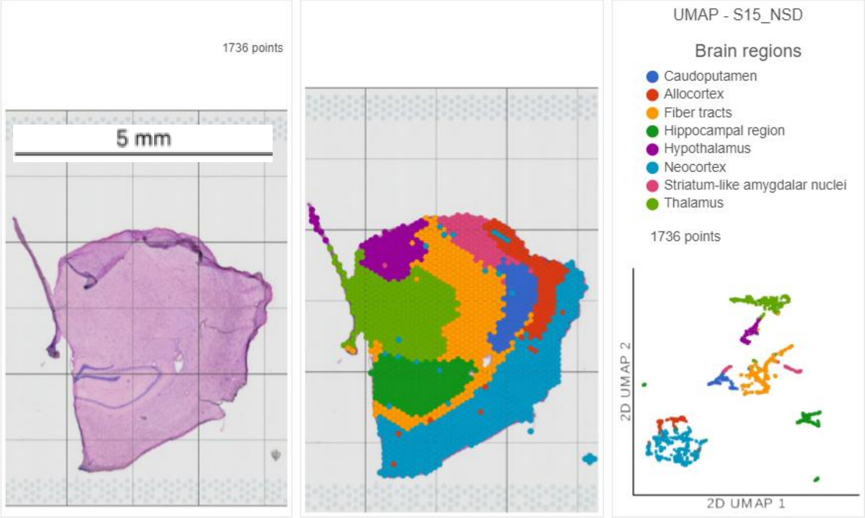

Sample 16

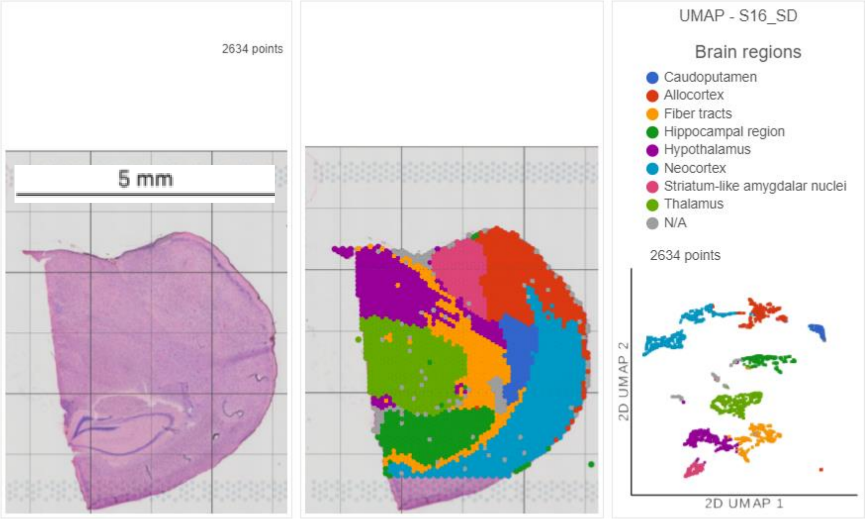


**Sup. Fig. 2. Supervised annotation of layers based on cytoarchitecture and known marker gene expression, related to Figure 1.** For each of the 16 samples, from left to right, coronal tissue section H&E histology staining, graph-based cluster identification at the spot level (each spot is color-coded based on the transcriptional signature computed from 20 principal components using Louvain clustering algorithm; the brain regions are labeled in the colored legend), and UMAP plot based on the transcriptional signature of each spot.

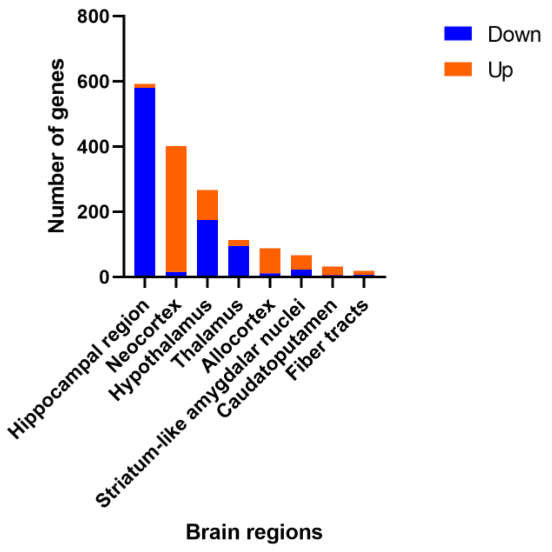


**Sup. Fig. 3. Number of up and down regulated DEGs in each brain region.** The x-axis represents the brain region and the y-axis indicates the count of significant DEGs. The height of each segment represents the number of significant DEGs in the respective category: red for upregulated DEGs and blue for downregulated DEGs. The total height of each bar represents the total number of significant DEGs in that brain region.

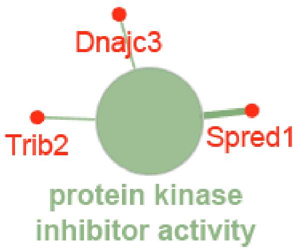


**Sup. Fig. 4. GO-molecular function (GO:MF) enrichment analysis for the 35 DEGs in common between the neocortex and allocortex.** Out of the 35 common DEGs, 3 upregulated genes were enriched for the "protein kinase inhibitor activity" molecular function.

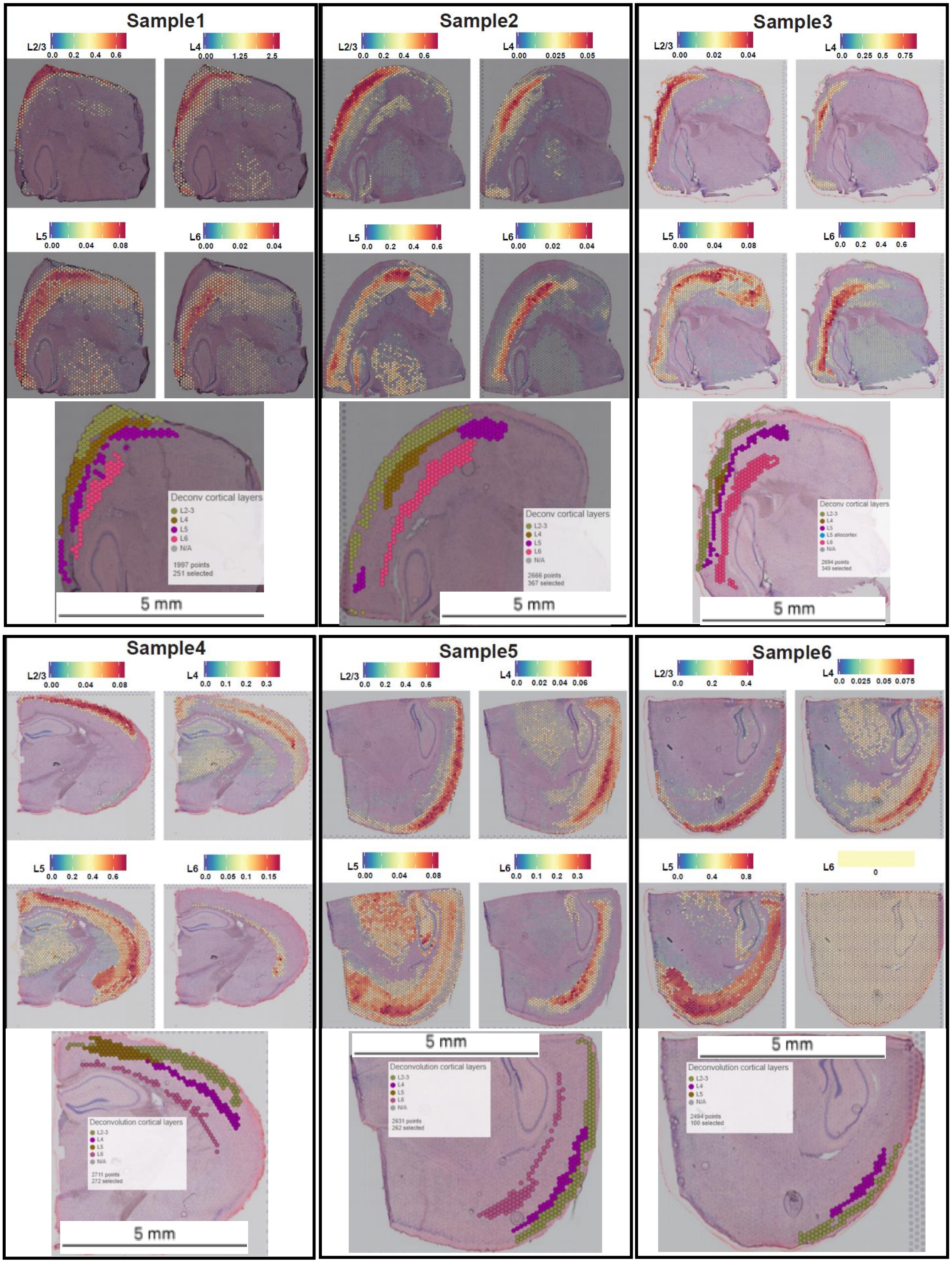
Sample1
L2/3
0.0 0.2 0.4 0.6
L4
0.00 1.25 2.5
L5
0.00 0.04 0.08
L6
0.00 0.02 0.04
Deconv cortical layers
L2-3
L4
L5
L6
N/A
1997 points
251 selected
5 mm
Sample2
L2/3
0.0 0.2 0.4 0.6
L4
0.00 0.025 0.05
L5
0.0 0.2 0.4 0.6
L6
0.00 0.02 0.04
Deconv cortical layers
L2-3
L4
L5
L6
N/A
2666 points
367 selected
5 mm
Sample3
L2/3
0.00 0.02 0.04
L4
0.0 0.25 0.5 0.75
L5
0.00 0.04 0.08
L6
0.0 0.2 0.4 0.6
Deconv cortical layers
L2-3
L4
L5
L5 allocortex
L6
N/A
2694 points
349 selected
5 mm
Sample4
L2/3
0.00 0.04 0.08
L4
0.0 0.1 0.2 0.3
L5
0.0 0.2 0.4 0.6
L6
0.0 0.05 0.1 0.15
Deconvolution cortical layers
L2-3
L4
L5
L6
N/A
2711 points
272 selected
5 mm
Sample5
L2/3
0.0 0.2 0.4 0.6
L4
0.0 0.02 0.04 0.06
L5
0.00 0.04 0.08
L6
0.0 0.1 0.2 0.3
5 mm
Deconvolution cortical layers
L2-3
L4
L5
L6
N/A
2631 points
262 selected
Sample6
L2/3
0.0 0.2 0.4
L4
0.0 0.025 0.05 0.075
L5
0.0 0.4 0.8
L6
0
5 mm
Deconvolution cortical layers
L2-3
L4
L5
N/A
2494 points
100 selected

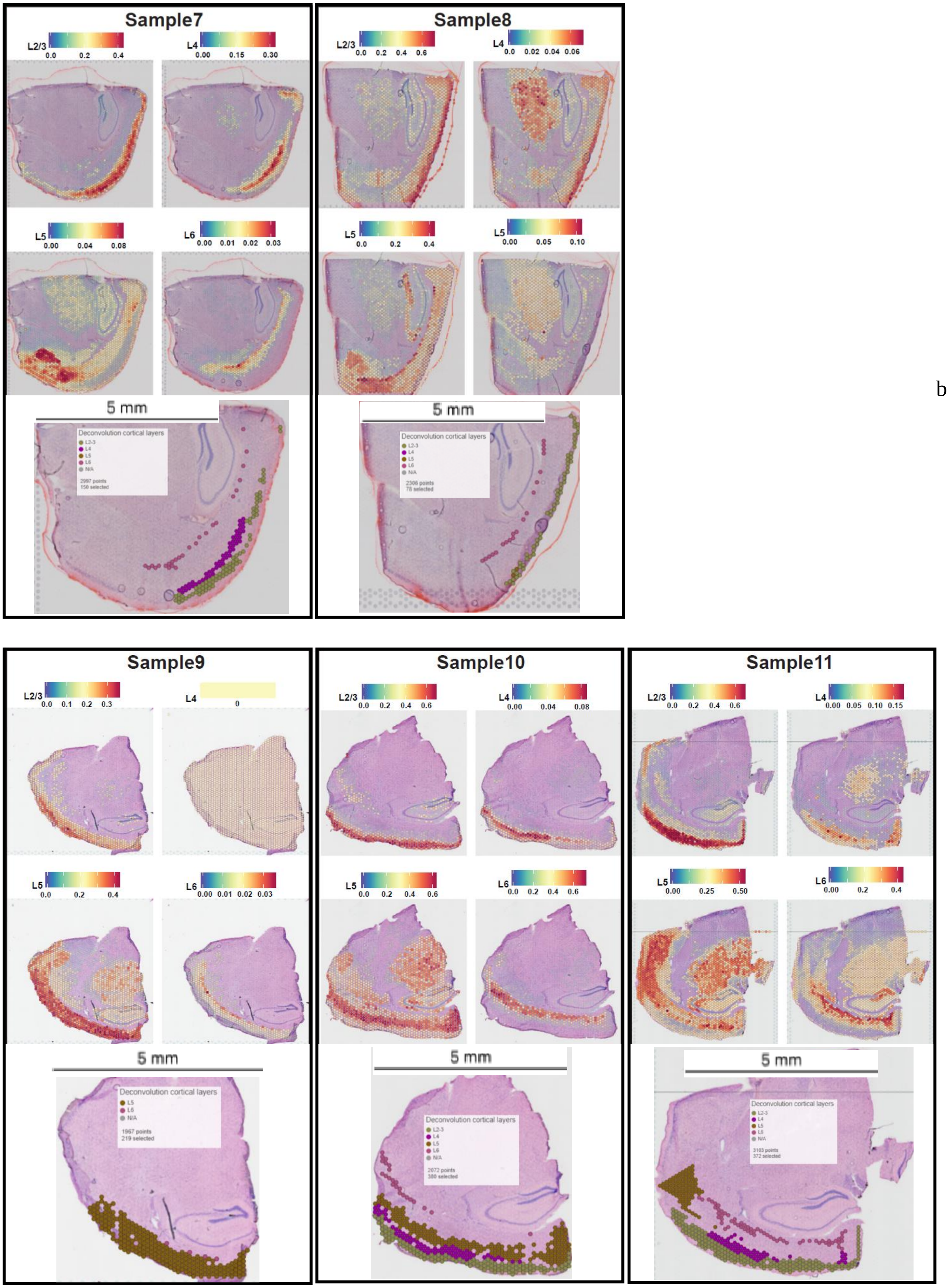

Sample7
L2/3
0.0 0.2 0.4
L4
0.00 0.15 0.30
L5
0.00 0.04 0.08
L6
0.00 0.01 0.02 0.03
5 mm
Deconvolution cortical layers
Sample8
L2/3
0.0 0.2 0.4 0.6
L4
0.0 0.02 0.04 0.06
L5
0.0 0.2 0.4
L5
0.00 0.05 0.10
5 mm
Deconvolution cortical layers
Sample9
L2/3
0.0 0.1 0.2 0.3
L4
0
L5
0.0 0.2 0.4
L6
0.00 0.01 0.02 0.03
5 mm
Deconvolution cortical layers
Sample10
L2/3
0.0 0.2 0.4 0.6
L4
0.00 0.04 0.08
L5
0.0 0.2 0.4 0.6
L6
0.0 0.2 0.4 0.6
5 mm
Deconvolution cortical layers
Sample11
L2/3
0.0 0.2 0.4 0.6
L4
0.00 0.05 0.10 0.15
L5
0.00 0.25 0.50
L6
0.00 0.2 0.4
5 mm
Deconvolution cortical layers


b

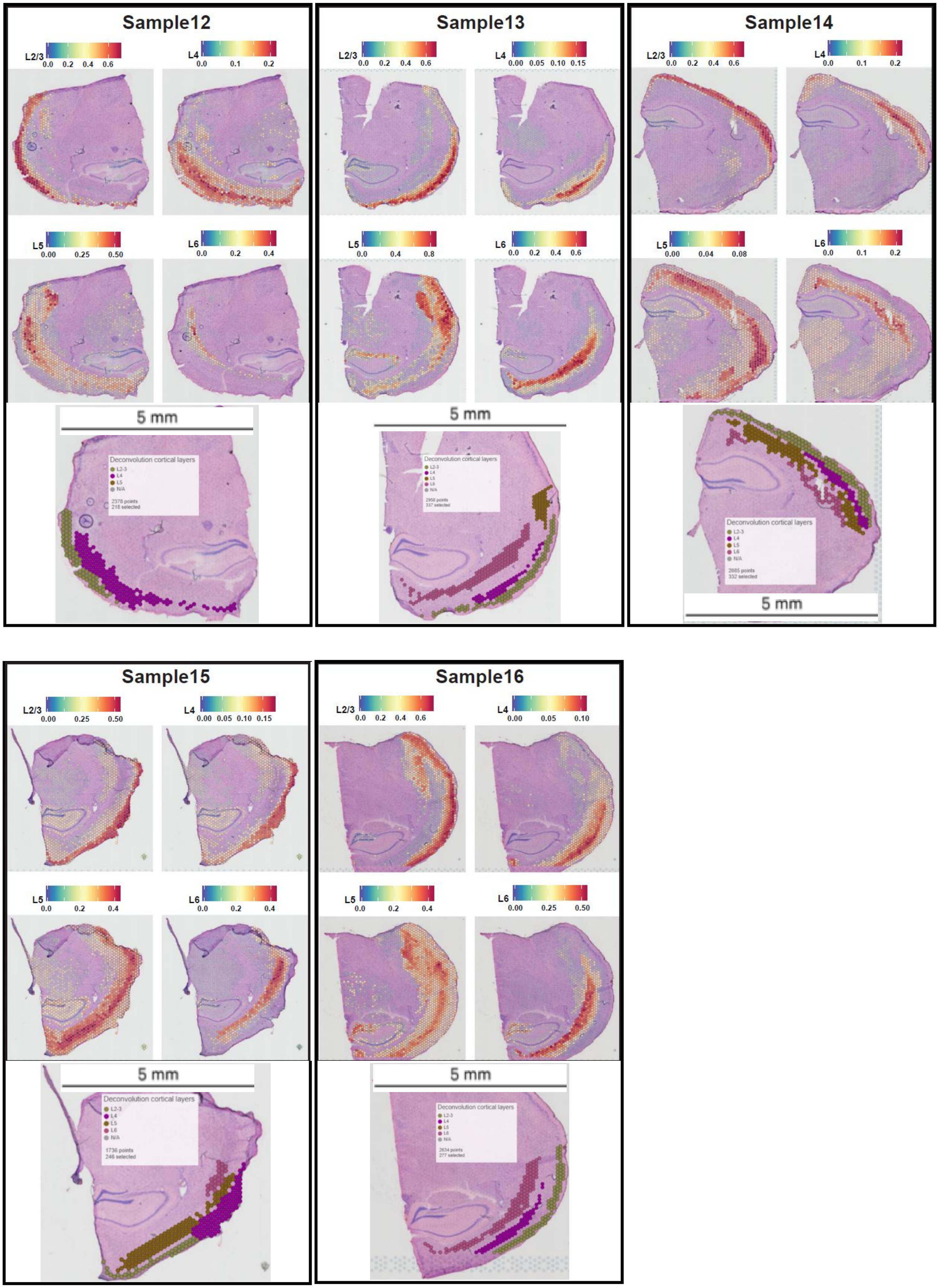


**Sup. Fig. 5. Deconvolution of cortical layers.** Deconvolution of cortical layers for all 16 samples. We used a reference scRNA-seq dataset of ~14,000 adult mouse cortical cell taxonomy from the Allen Institute

(https://www.nature.com/articles/nn.4216). Prediction scores are represented at the top of each panel ranging from blue (low confidence) to red (high confidence ). At the bottom of each sample is an aggregation of the deconvoluted cortical layers (green = layer 2/3, purple = layer 4, brown = layer 5, pink = layer 6).

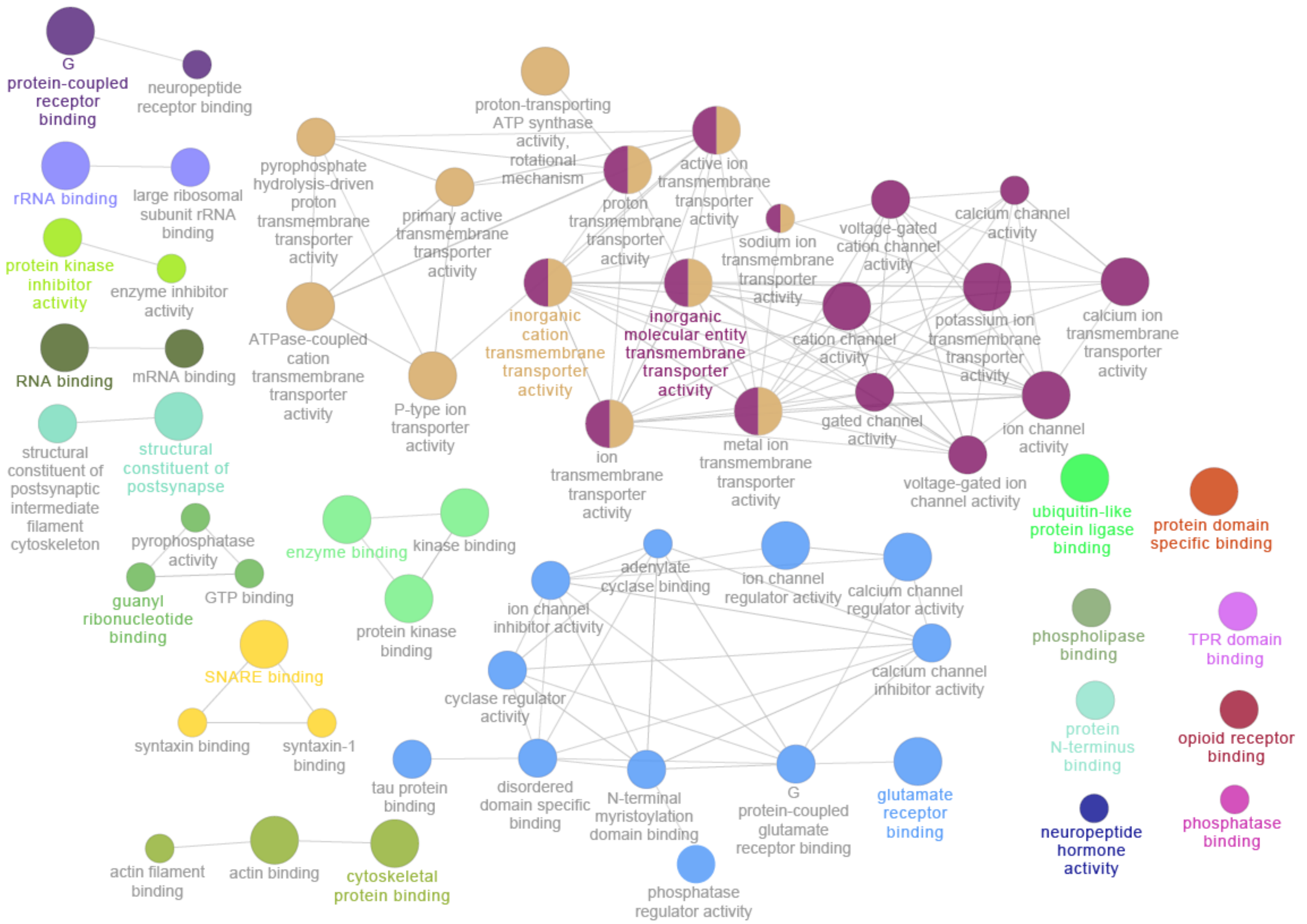


**Sup. Fig. 6. GO-molecular function (GO:MF) enrichment analysis of the 413 after registration in Allen common space.** Molecular functions enriched from the significant DEGs after registration in Allen common space. A gene is significant if its FDR step-up < 0.001 and its log2fold-change ≥ |0.2|. The size of the circle for each enriched molecular function is proportional to the significance. Only molecular functions with a corrected p-value < 0.05 are displayed (two-sided hypergeometric test, Bonferroni step down).

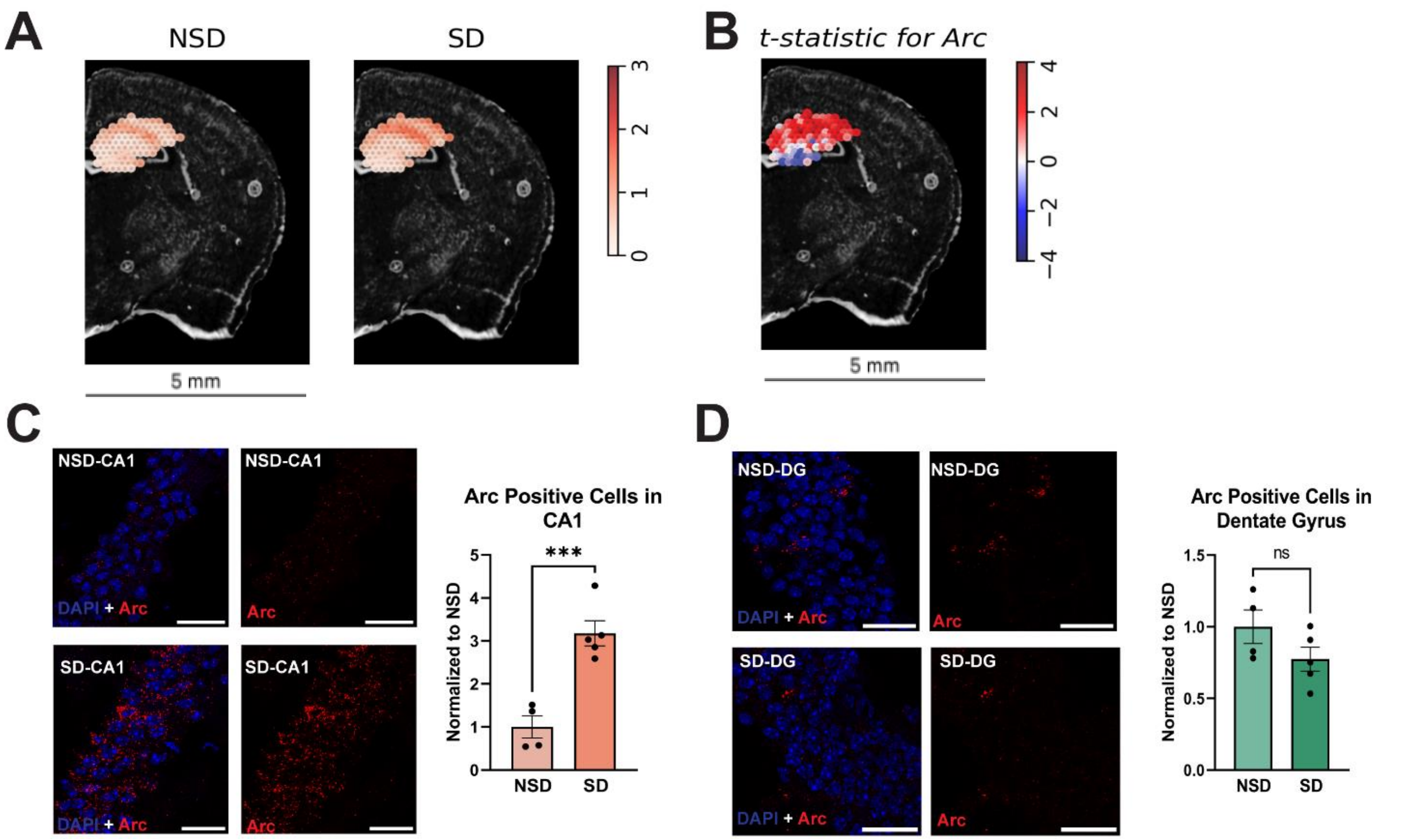


**Sup. Fig. 7. Hippocampal RNAscope validates the analysis of Arc expression determined by STANLY.**

**A.** Mean of the log base 2 normalized gene count per digital spot for the NSD (n=6) group on the left and SD (n=7) on the right in the common Allen space. The range of the color bar for the mean calculations is set from 0 (white) to a log2fold-change of 3 (red). **B**. The SD>NSD t-statistic for the log2fold change. The t-statistic color bar is bounded to [-4,4], which is approximately equal to the corrected FDR $p < 0.1$. **C**. RNAscope measure of Arc in the CA1. Example images provided for NSD (n=4, top panels) and SD (n=5, bottom panels). DAPI (blue) + Arc (Red) on the left, and Arc channel alone on the right. Arc positive cells were counted and quantified as the proportion of positive cells in the region and reported in the bar graph as the fold change relative to the NSD condition (mean ± standard error). SD (3.18 ± 0.29) is significantly higher than NSD (1.00 ± 0.26) as determined by two-tailed t-test ($t(7) = 5.44$, $p = 0.001$). **D**. RNAscope analysis of the dentate gyrus (DG). Example images provided for NSD (n=4, top panels) and SD (n=5 bottom panels). DAPI (blue) + Arc (Red) on the left, and Arc channel alone on the right. Arc positive cells were counted and quantified as the proportion of positive cells in the region and reported in the bar graph as the fold change relative to the NSD

condition. SD (0.77 ± 0.08) is not significantly different from NSD (1.00 ± 0.12) as determined by two-tailed t-test (t(7) = 1.62, p = 0.149). The scale bar in each RNAscope image is 25 μm.

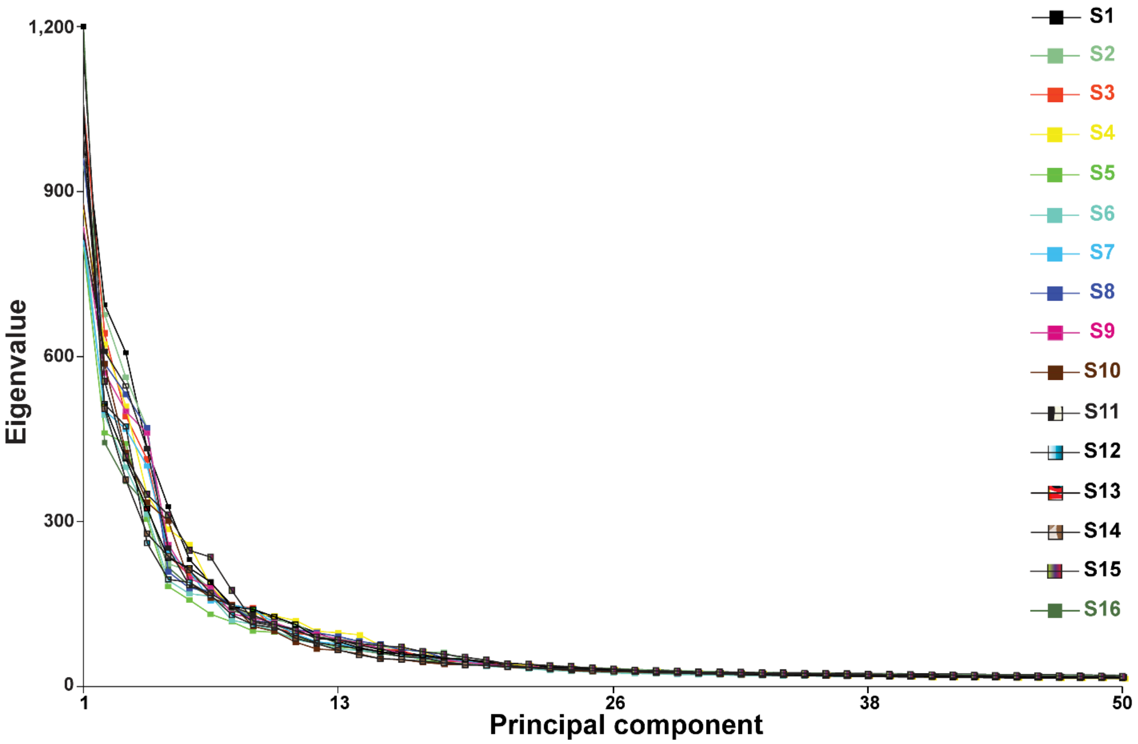


**Sup. Fig. 8. Determining the dimensionality of the dataset.** Scree plot ("elbow" plot) with X-axis representing principal components (PCs) and Y-axis representing eigenvalues. This shows how much variation each PC represents and is used to determine the number of principal components to retain for downstream analysis, including UMAP and graph-base clustering. The "elbow" point of the graph where the eigenvalues levels off around 20 PCs is considered as a cutoff point for downstream analysis.

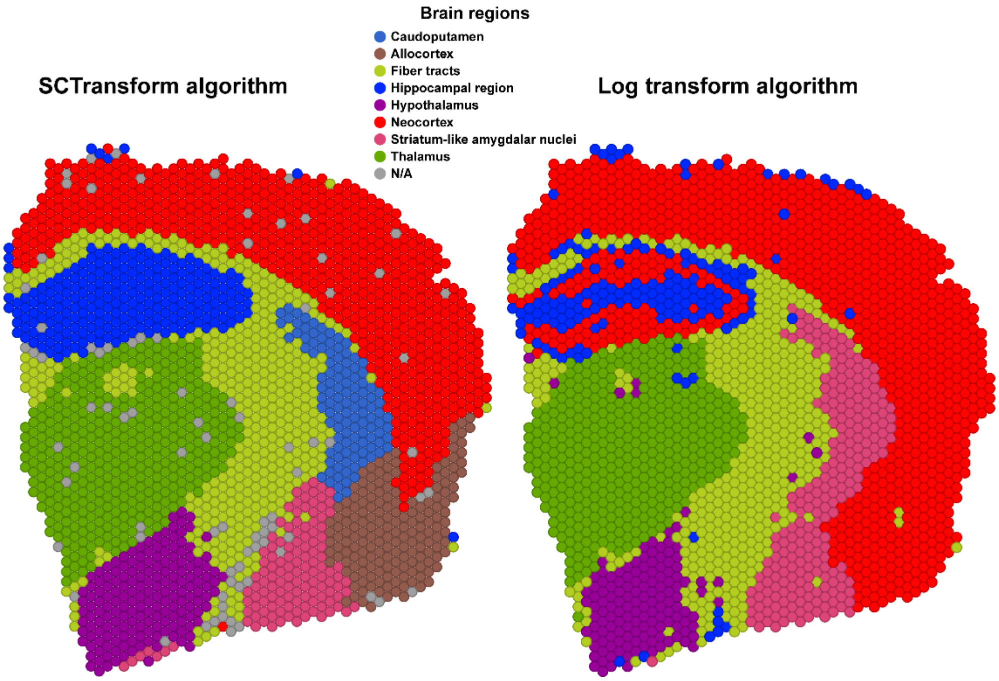


**Sup. Fig. 9. The SCTransform algorithm yields a more accurate representation of the different brain regions.** Comparison of two normalization algorithms to identify brain regions. The SCTransform algorithm (left) identifies two subregions of the striatum (caudatoputamen and striatum-like amygdalar nuclei) as well as two subregions in the cortex (neocortex and allocortex) which is not the case with the log-transformed algorithm (right). In addition, with SCTransform, there are no spots in the hippocampal region identified as part of the neocortex.

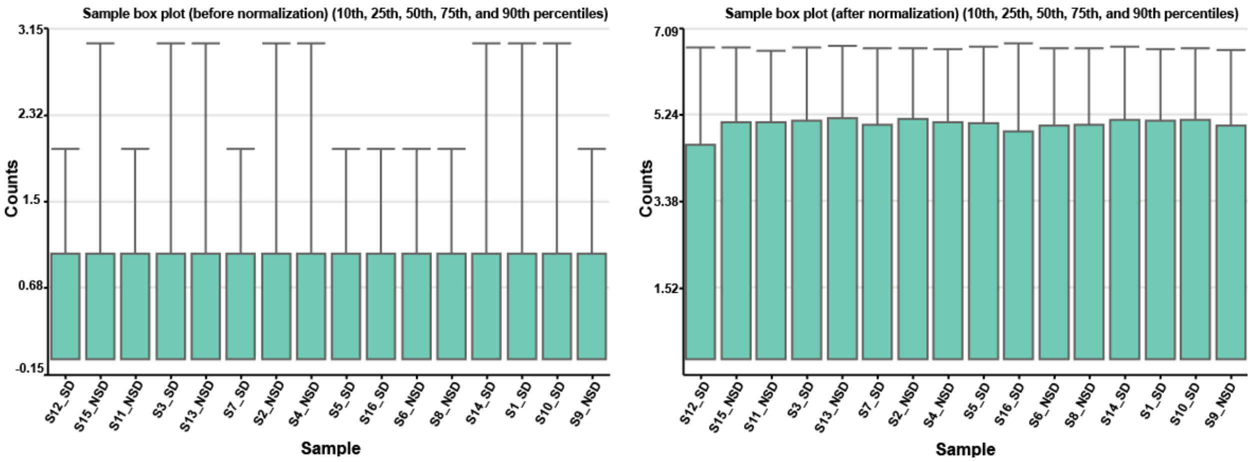


**Sup. Fig. 10. Box plots illustrating the distribution of gene expression levels before (left) and after (right) normalization across samples.** The y-axis represents the gene expression values (log normalized counts). The x-axis denotes the different samples. The box itself spans to the 75th percentile, encompassing the upper interquartile range. These box plots highlight the effect of normalization on the gene expression data, providing valuable information for assessing the quality and comparability of gene expression profiles across different samples.

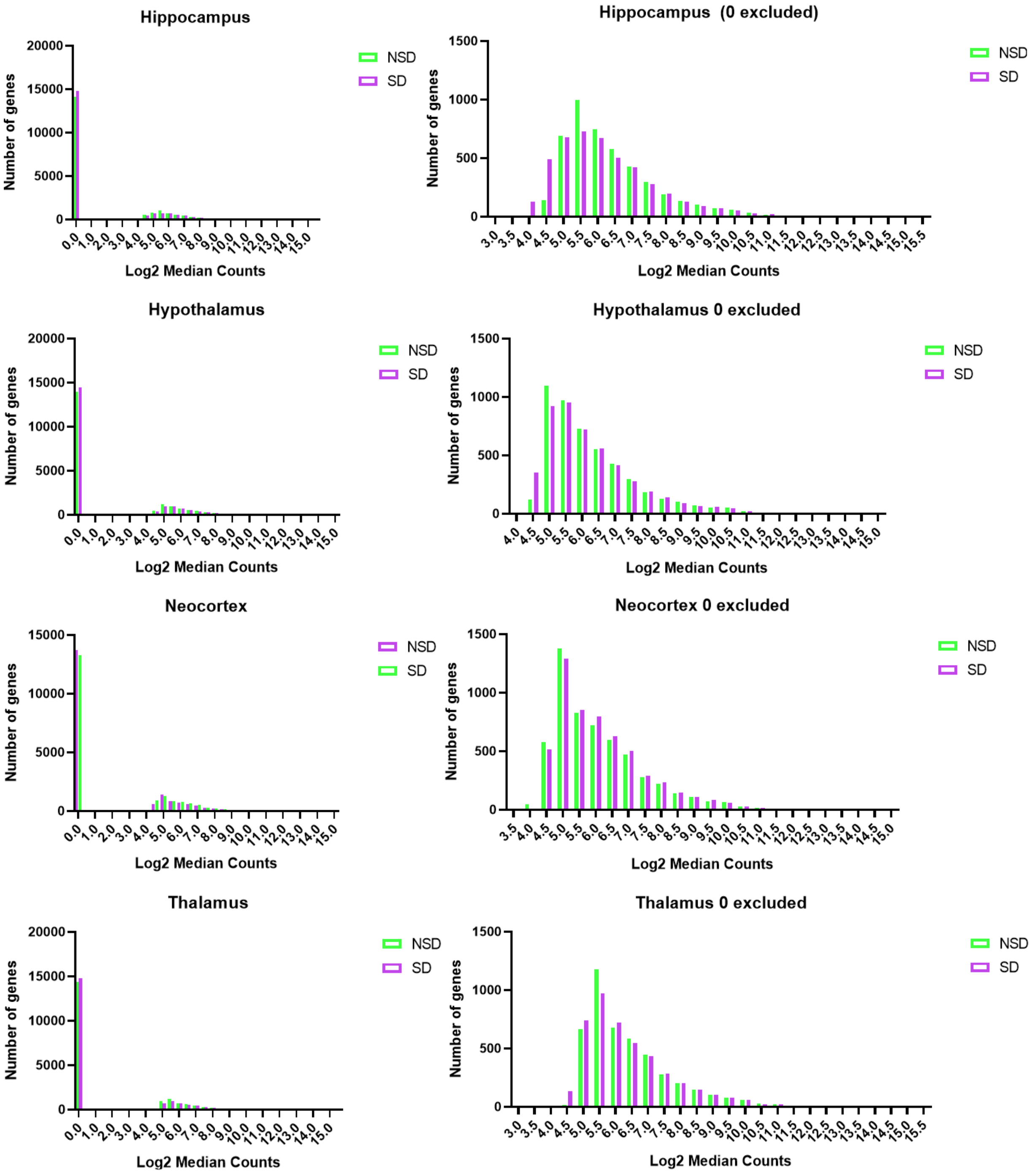


**Sup. Fig. 11. Distribution of median counts (log2 base) in the four most affected brain regions after sleep deprivation.** For each brain region, the histogram on the left represents the distribution of all median count values, whereas the histogram on the right represents the distribution of median count values after excluding the 0 median counts. The non-parametric Kruskal-Wallis rank sum test assigns a log2 median count of 0 for the genes that are not expressed in the brain region.

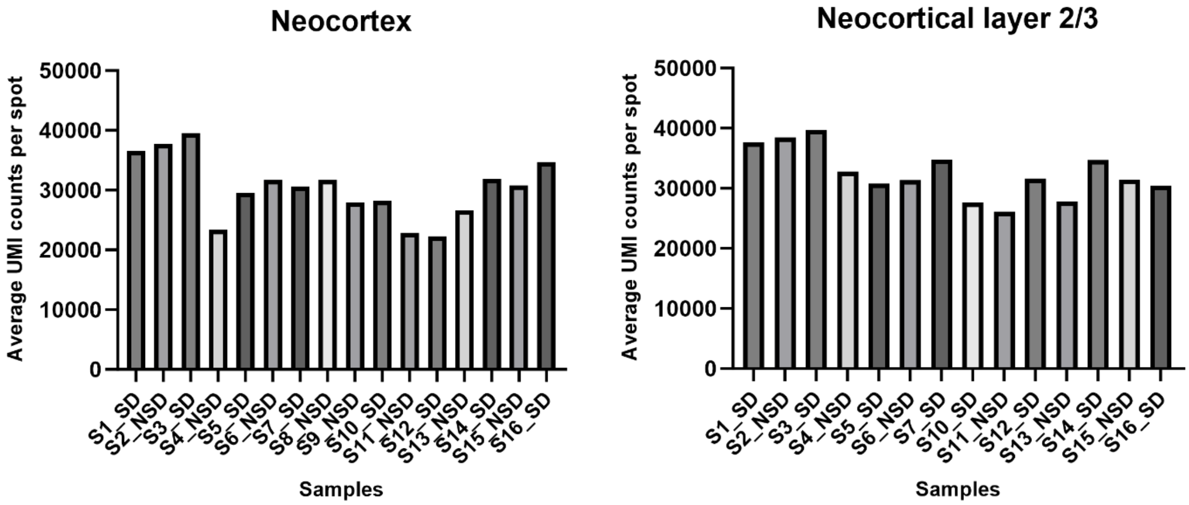


**Sup. Fig. 12. Average UMI counts per spot in neocortex and neocortical layer 2/3.** For each sample, the histograms represent the average UMI counts per spot within the neocortex (left) or within the specific neocortical layer 2/3 (right). This demonstrates a maintained sequenced depth across samples within a brain region or subregion.

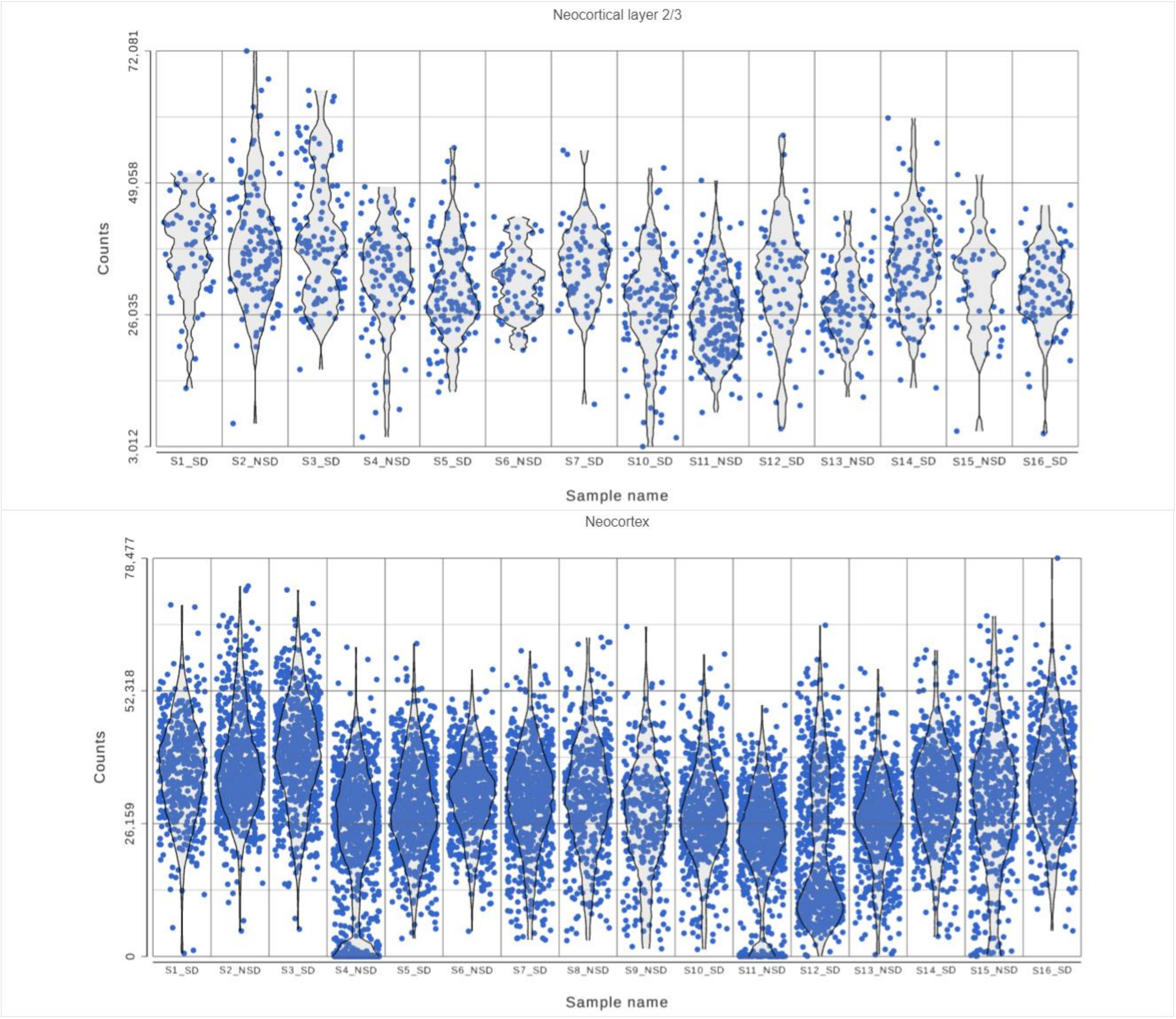


**Sup. Fig. 13. Distribution of UMI counts per spot in neocortex and neocortical layer 2/3.** For each sample, the violin plots represent the variability of UMI counts of each spot within the neocortex (top) or within the specific neocortical layer 2/3 (bottom). This shows a negligible variability of counts from sample to sample.

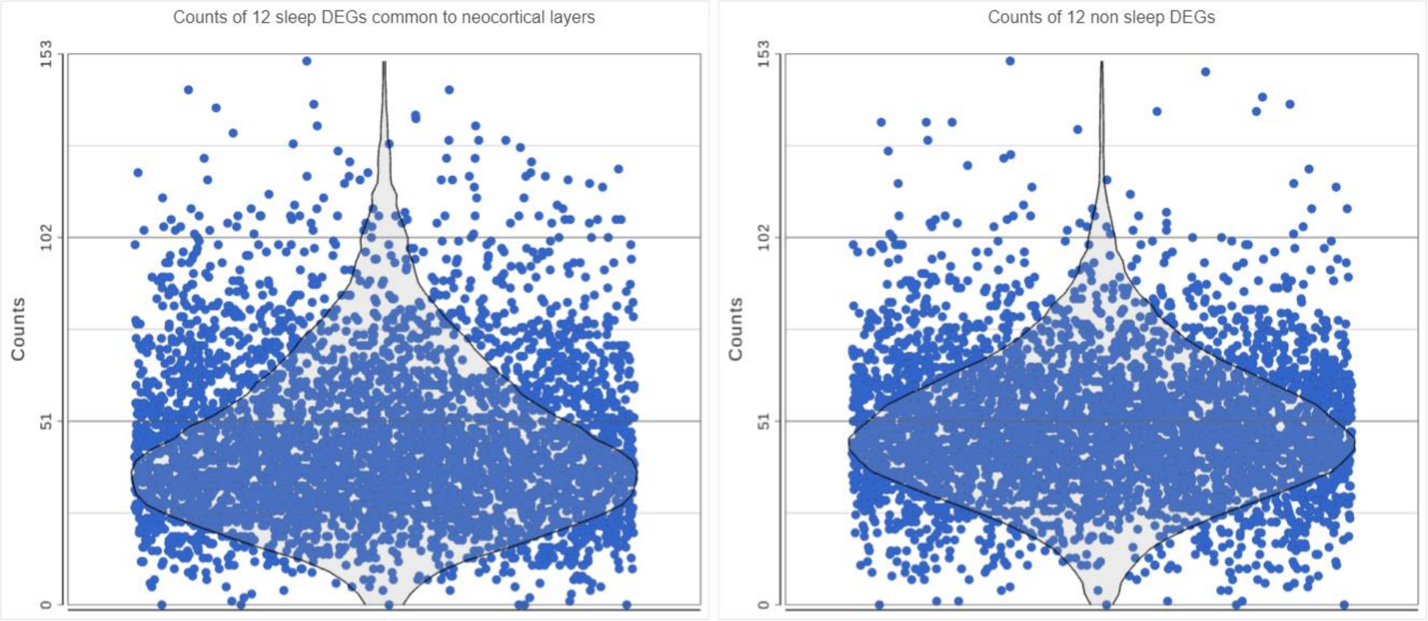


**Sup. Fig. 14. Distribution of UMI counts of sleep DEGs vs non sleep DEGs.** There are 12 DEGs commonly affected after sleep deprivation in each neocortical layer. When comparing the UMI variability of these 12 DEGs (left) with that of 12 genes unaffected by sleep deprivation (right) in the neocortical layers, no difference is observed in UMI variability.

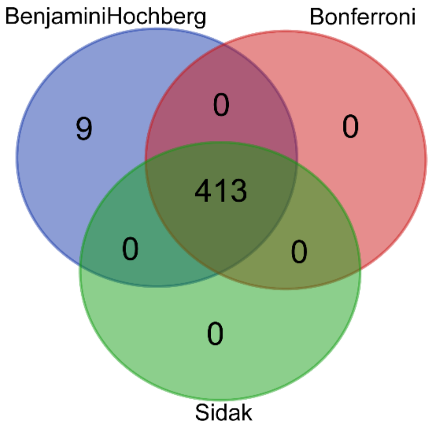


**Sup. Fig. 15. Overlap of DEGs between three different statistical models.** The diagram shows the number of significant genes differentially expressed in each statistical model (Benjamini-Hochberg, Bonferroni, and Sidak) as well as the number of genes shared between any of them. The overlapping region in the center represents genes significantly changed in all three statistical models, with Bonferroni and Sidak corrections demonstrating 100% overlap.
