# Supplementary material for: Spatial transcriptomics reveals unique gene expression changes in different brain regions after sleep deprivation
Source: Nat Commun. 2023 Nov 4;14:7095. doi: 10.1038/s41467-023-42751-z (PMC10625558; doi:10.1038/s41467-023-42751-z)
Supplement: Supplementary file 3 — Description of Additional Supplementary Files [file 41467_2023_42751_MOESM3_ESM.pdf]

Description of Additional Supplementary Files

File Name: Supplementary Data 1
Description: This table presents quality control metrics, including data from the web summaries (HTML files) generated by the 10X Genomics Space Ranger pipeline. These metrics offer insights into the quality and characteristics of the spatial transcriptomic sequencing data. Additionally, this table provides information about the age and sex of the mice used in the study.

File Name: Supplementary Data 2
Description: This table displays the results of the differential gene expression analysis conducted after sleep deprivation in various brain regions. The statistical model used for this analysis was a Rank-Sum Test Kruskal-Wallis, a non-parametric statistical test. The median gene expression was calculated for each gene across all the spots within the brain regions. Fold change was determined as the estimated median expression at the sleep deprivation condition divided by the estimated median expression at the non-sleep deprived condition. False Discovery Rate (FDR) correction was applied to the p-values to account for multiple testing.

File Name: Supplementary Data 3
Description: This table showcases the genes that exhibit significant differential expression (FDR < 0.001 and Fold Change > |1.2|) following sleep deprivation in different brain regions. The statistical model used for this analysis was a Rank-Sum Test Kruskal-Wallis, a non-parametric statistical test. The median gene expression was calculated for each gene across all the spots within the brain regions. Fold change was determined as the estimated median expression at the sleep deprivation condition divided by the estimated median expression at the non-sleep deprived condition. False Discovery Rate (FDR) correction was applied to the p-values to account for multiple testing.

File Name: Supplementary Data 4
Description: This table contains the outputs of significantly enriched molecular functions enrichment analysis using ClueGO. The statistical model used for this analysis was a Two-sided hypergeometric test. Bonferroni correction was applied to the p-values to account for multiple testing. Only molecular function with a corrected p-value < 0.05 are displayed.

File Name: Supplementary Data 5
Description: This table lists the genes that are significantly differentially expressed (FDR < 0.001 and Fold Change > |1.2|) and overlap between two or more brain regions after sleep deprivation.

File Name: Supplementary Data 6
Description: This table presents the results of the differential gene expression analysis conducted after sleep deprivation specifically in deconvoluted hippocampal subregions. The statistical model used for this analysis was a Rank-Sum Test Kruskal-Wallis, a non-parametric statistical test. The median gene expression was calculated for each gene across all the spots within the hippocampal subregions. Fold change was calculated as the estimated median expression at the sleep deprivation condition divided by the estimated median

expression at the non-sleep deprived condition. False Discovery Rate (FDR) correction was then applied to the p-values to account for multiple testing.

File Name: Supplementary Data 7
Description: This table highlights genes that exhibit significant differential expression (FDR < 0.1 and Fold Change > |1.2|) after sleep deprivation in hippocampal subregions. The statistical model used for this analysis was a Rank-Sum Test Kruskal-Wallis, a non-parametric statistical test. The median gene expression was calculated for each gene across all the spots within the hippocampal subregions. Fold change was calculated as the estimated median expression at the sleep deprivation condition divided by the estimated median expression at the non-sleep deprived condition. False Discovery Rate (FDR) correction was then applied to the p-values to account for multiple testing.

File Name: Supplementary Data 8
Description: This table displays the results of the differential gene expression analysis conducted after sleep deprivation in different deconvoluted layers of the neocortex. The statistical model used for this analysis was a Rank-Sum Test Kruskal-Wallis, a non-parametric statistical test. The median gene expression was calculated for each gene across all the spots within the neocortical layers. Fold change was determined as the estimated median expression at the sleep deprivation condition divided by the estimated median expression at the non-sleep deprived condition. False Discovery Rate (FDR) correction was applied to the p-values to account for multiple testing.

File Name: Supplementary Data 9
Description: This table focuses on genes with significant differential expression (FDR < 0.001 and Fold Change > |1.2|) following sleep deprivation in neocortical layers. The statistical model used for this analysis was a Rank-Sum Test Kruskal-Wallis, a non-parametric statistical test. The median gene expression was calculated for each gene across all the spots within the neocortical layers. Fold change was determined as the estimated median expression at the sleep deprivation condition divided by the estimated median expression at the non-sleep deprived condition. False Discovery Rate (FDR) correction was applied to the p-values to account for multiple testing.

File Name: Supplementary Data 10
Description: This table provides the results of STANLY analysis, specifically examining significant differentially expressed genes following sleep deprivation at the individual spatial spots level. The statistical model used for this analysis was a two-tailed t-test. This test was applied to assess the significance of differential expressions between the sleep-deprived and non-sleep-deprived conditions at each individual spot.
