# Supplementary material for: Spatial transcriptomics reveals unique gene expression changes in different brain regions after sleep deprivation
Source: Nat Commun. 2023 Nov 4;14:7095. doi: 10.1038/s41467-023-42751-z (PMC10625558; doi:10.1038/s41467-023-42751-z)
Supplement: Supplementary file 14 — Reporting Summary [file 41467_2023_42751_MOESM14_ESM.pdf]

nature portfolio

Corresponding author(s): Ted Abel and Thomas Nickl-Jockschat

Last updated by author(s): Oct 6, 2023

Reporting Summary

Nature Portfolio wishes to improve the reproducibility of the work that we publish. This form provides structure for consistency and transparency in reporting. For further information on Nature Portfolio policies, see our Editorial Policies and the Editorial Policy Checklist.

Statistics

For all statistical analyses, confirm that the following items are present in the figure legend, table legend, main text, or Methods section.

n/a	Confirmed	
☐	☒	The exact sample size (*n*) for each experimental group/condition, given as a discrete number and unit of measurement
☐	☒	A statement on whether measurements were taken from distinct samples or whether the same sample was measured repeatedly
☐	☒	The statistical test(s) used AND whether they are one- or two-sided<br>*Only common tests should be described solely by name; describe more complex techniques in the Methods section.*
☐	☒	A description of all covariates tested
☐	☒	A description of any assumptions or corrections, such as tests of normality and adjustment for multiple comparisons
☐	☒	A full description of the statistical parameters including central tendency (e.g. means) or other basic estimates (e.g. regression coefficient) AND variation (e.g. standard deviation) or associated estimates of uncertainty (e.g. confidence intervals)
☐	☒	For null hypothesis testing, the test statistic (e.g. *F*, *t*, *r*) with confidence intervals, effect sizes, degrees of freedom and *P* value noted<br>*Give P values as exact values whenever suitable.*
☒	☐	For Bayesian analysis, information on the choice of priors and Markov chain Monte Carlo settings
☒	☐	For hierarchical and complex designs, identification of the appropriate level for tests and full reporting of outcomes
☒	☐	Estimates of effect sizes (e.g. Cohen's *d*, Pearson's *r*), indicating how they were calculated

Our web collection on statistics for biologists contains articles on many of the points above.

Software and code

Policy information about availability of computer code

Data collection	Leica cryostat, Olympus BX61 Upright Microscope, Olympus, Illumina NovaSeq 6000 SP, Leica SP8 microscope
Data analysis	Visium imaging: Olympus CellSens software v.3.2<br>Visium data processing: Space Ranger v.1.3.1, STAR v.2.7.10a, Partek Flow package (Build version 10.0.21.0621), Cytoscape 3.9.0 software<br>STANLY: SimpleITK v.5.3.0, scikit-image v.0.19.3, SciPy v.1.7.3, NumPy v.1.21.5<br><br>The code for the deconvolution analysis can be accessed through GitHub (https://github.com/YannVRB/Sleep-deprivation-spatial-transcriptomic.git). The code for STANLY and subsequent analysis can be accessed through GitLab (https://research-git.uiowa.edu/zjpeters/STANLY).

For manuscripts utilizing custom algorithms or software that are central to the research but not yet described in published literature, software must be made available to editors and reviewers. We strongly encourage code deposition in a community repository (e.g. GitHub). See the Nature Portfolio guidelines for submitting code & software for further information.

Data

Policy information about availability of data

All manuscripts must include a data availability statement. This statement should provide the following information, where applicable:

- Accession codes, unique identifiers, or web links for publicly available datasets
- A description of any restrictions on data availability
- For clinical datasets or third party data, please ensure that the statement adheres to our policy

The spatial RNA-seq data has been deposited in the National Center for Biotechnology Information (NCBI) Gene Expression Omnibus (GEO) under accession number GSE222410.
Two screenshots of the reference mouse Allen brain atlas (http://atlas.brain-map.org) were used in two figures. The coronal section image 70 was used in Figure 1C. The coronal section image 70 from the Allen P56 Mouse Common Coordinate Framework (CCF) was used in Figure 5A3 as well as a template for the alignment step in STANLY.
A reference scRNA-seq dataset of ~14,000 adult mouse cortical cell taxonomy from the Allen Institute was used for deconvolution (DOI: 10.1038/nn.4216).
Mm10 reference genome for spatial transcriptomic: https://cf.10xgenomics.com/supp/spatial-exp/refdata-gex-mm10-2020-A.tar.gz

Research involving human participants, their data, or biological material

Policy information about studies with human participants or human data. See also policy information about sex, gender (identity/presentation), and sexual orientation and race, ethnicity and racism.

Reporting on sex and gender	N/A
Reporting on race, ethnicity, or other socially relevant groupings	N/A
Population characteristics	N/A
Recruitment	N/A
Ethics oversight	N/A

Note that full information on the approval of the study protocol must also be provided in the manuscript.

Field-specific reporting

Please select the one below that is the best fit for your research. If you are not sure, read the appropriate sections before making your selection.

☒ Life sciences ☐ Behavioural & social sciences ☐ Ecological, evolutionary & environmental sciences

For a reference copy of the document with all sections, see nature.com/documents/nr-reporting-summary-flat.pdf

Life sciences study design

All studies must disclose on these points even when the disclosure is negative.

Sample size	Sample sizes were estimated based on previous studies in the field (Schurch, N. J. et al. RNA 22, 839-851 (2016), Liu, Y. et al. Bioinformatics 30, 301-304 (2014)). No statistical methods were used to predetermine sample size.
Data exclusions	Two samples were excluded during the identification of neocortical layers 2/3 due to inaccuracies in the deconvolution algorithm, which hindered the prediction of the cell type of interest at individual spots.<br>Two samples were excluded during the identification of neocortical layers 4 due to inaccuracies in the deconvolution algorithm, which hindered the prediction of the cell type of interest at individual spots.<br>Five samples were excluded during the identification of neocortical layers 5 due to inaccuracies in the deconvolution algorithm, which hindered the prediction of the cell type of interest at individual spots.<br>Three samples were excluded during the identification of neocortical layers 6 due to inaccuracies in the deconvolution algorithm, which hindered the prediction of the cell type of interest at individual spots.<br>Two samples were excluded during the identification of hippocampal subregions because of issue damage or a large amount of tissue folding after adhesion to the slide.<br>Three samples were excluded during data preprocessing for STANLY because of tissue damage or a large amount of tissue folding after adhesion to the slide.
Replication	We performed sleep deprivation from two independent cohorts and processed the samples for spatial transcriptomic independently with a batch correction applied between the two cohorts.<br>To validate the reliability of our STANLY analyses, using samples from independent sleep deprivation experiments, we performed in situ hybridization using RNAscope (ACD) for Arc expression in the hippocampus.

Randomization	In each experimental batch, animals were chosen randomly based on their age. No software for randomization were used. Sleep deprived mice were cage-mates of the mice in the non-sleep deprived condition.
Blinding	Spatial transcriptomic RNA was extracted by experimenter blind to the treatment groups. Sections for spatial gene expression were prepared blindly. The investigators were blinded to the treatment groups during data analysis until the differential gene expression analysis stage. At this point, they had to set up parameters and assign treatments to each sample to distinguish between sleep-deprived and non-sleep-deprived animals.

Reporting for specific materials, systems and methods

We require information from authors about some types of materials, experimental systems and methods used in many studies. Here, indicate whether each material, system or method listed is relevant to your study. If you are not sure if a list item applies to your research, read the appropriate section before selecting a response.

Materials & experimental systems

n/a	Involved in the study	
☒	☐	Antibodies
☒	☐	Eukaryotic cell lines
☒	☐	Palaeontology and archaeology
☐	☒	Animals and other organisms
☒	☐	Clinical data
☒	☐	Dual use research of concern
☒	☐	Plants

Methods

n/a	Involved in the study	
☒	☐	ChIP-seq
☒	☐	Flow cytometry
☒	☐	MRI-based neuroimaging

Animals and other research organisms

Policy information about studies involving animals; ARRIVE guidelines recommended for reporting animal research, and Sex and Gender in Research

Laboratory animals	In this study, Male C57BL/6J mice (Jackson Laboratory #000664), age 2.5-3.5 months were used.
Wild animals	The study did not include any wild animals.
Reporting on sex	Only male mice were included.
Field-collected samples	The study did not include any samples collected at the field.
Ethics oversight	All experiments detailed herein complied with the regulations formulated by the Institutional Animal Care and Use Committee (IACUC) of the University of Iowa.

Note that full information on the approval of the study protocol must also be provided in the manuscript.
